# Association Between Type I and II Diabetes With Gallbladder Stone Disease

**DOI:** 10.3389/fendo.2018.00720

**Published:** 2018-11-29

**Authors:** Chien-Hua Chen, Cheng-Li Lin, Chung-Y. Hsu, Chia-Hung Kao

**Affiliations:** ^1^Digestive Disease Center, Changbing Show-Chwan Memorial Hospital, Changhua, Taiwan; ^2^Digestive Disease Center, Show-Chwan Memorial Hospital, Changhua, Taiwan; ^3^Department of Food Science and Technology, Hungkuang University, Taichung, Taiwan; ^4^Chung Chou University of Science and Technology, Changhua, Taiwan; ^5^Management Office for Health Data, China Medical University Hospital, Taichung, Taiwan; ^6^College of Medicine, China Medical University, Taichung, Taiwan; ^7^Graduate Institute of Clinical Medical Science, China Medical University, Taichung, Taiwan; ^8^Graduate Institute of Biomedical Sciences and School of Medicine, College of Medicine, China Medical University, Taichung, Taiwan; ^9^Department of Nuclear Medicine and PET Center, China Medical University Hospital, Taichung, Taiwan; ^10^Department of Bioinformatics and Medical Engineering, Asia University, Taichung, Taiwan

**Keywords:** type 2 diabetes, gallbladder stone disease, type 1 diabetes, cohort study, ICD-9 codes

## Abstract

**Objective:** To assess the association of type 1 diabetes (T1DM) and type 2 diabetes (T2DM) with the subsequent development of gallbladder stone disease (GSD).

**Setting:** Cohort Study.

**Participants:** We identified two study cohort groups to evaluate the association of T1DM and T2DM with the development of GSD. The first group comprised a T1DM cohort of 7015 patients aged ≤ 40 years and a non-diabetes cohort randomly matched with the study cohort (4:1). The second group comprised a T2DM cohort of 51,689 patients aged ≥20 years and a non-diabetes cohort randomly matched with the study cohort (1:1). All patients were studied from 1996 to the end of 2011 or withdrawal from the National Health Insurance program to determine the incidence of GSD.

**Results:** Compared with patients without diabetes, those with T1DM had a decreased risk of GSD [adjusted hazard ratios (aHR) = 0.48, 95% confidence interval (CI) = 0.25–0.92]. Those with T2DM had an increased risk of GSD (aHR = 1.55, 95% CI = 1.41–1.69), after adjustment for age, sex, comorbidities, and number of parity. The relative risk of GSD in the T2DM cohort was higher than that in the non-diabetes cohort in each group of age, sex, and patients with or without comorbidity. However, the relative risk of GSD in the T1DM cohort was lower than that in the non-diabetes cohort only in the age group of 20–40 years.

**Conclusion:** Our population-based cohort study reveals a strong association between T2DM and GSD. However, an inverse relationship exists between T1DM and GSD in patients aged 20–40 years.

## Introduction

Gallbladder stone disease (GSD), characterized by crystalline deposits in the gallbladder, affects approximately 5–25% of the adult population globally (1, 2). GSD is the most common gastrointestinal disease in outpatient departments, and it can be easily ascertained by ultrasonography with an accuracy of approximately 90% (3). Reports have revealed that approximately 2–4% of patients with GSD develop symptoms each year; the annual incidence rates of acute cholecystitis, acute pancreatitis, and obstructive jaundice are 0.3–0.4%, 0.04–1.5%, and 0.1–0.4%, respectively (4–8). Moreover, nearly 20% of patients with GSD die after a first attack of acute pancreatitis or acute cholangitis (9). A report revealed that approximately 1–2% of patients with GSD require surgery each year, which places a heavy burden on medical expenditure and personnel levels (10). Therefore, identifying the risk of GSD to develop a program for the analysis and prevention of its potential complications is important.

Diabetes is one of the most common diseases in the world, and it has boosted public health concerns due to its complications such as retinopathy, nephropathy, neuropathy, ischemic heart disease, and peripheral vascular disease. The reported global prevalence of diabetes in 2013 was 382 million people, which is expected to increase to 592 million people by 2035 (11). Type 1 diabetes (T1DM) and type 2 diabetes (T2DM) are the main subtypes of diabetes, with 15% of patients with diabetes having T1DM and 85% having T2DM (11). Insulin resistance (IR) is the main pathogenesis for T2DM, and T2DM is a well-established risk factor for the development of GSD through reduced bile salt secretion and impaired gallbladder emptying (12–15). However, Insulin deficiency is the main pathogenesis for T1DM and the association between T1DM and GSD remains debated (16–18). Hence, we conducted a nationwide, population-based cohort study by analyzing data from Taiwan's National Health Insurance Research Database (NHIRD) to assess the association of T1DM and T2DM with the subsequent development of GSD.

## Methods

### Patient and public involvement

**Figure 1:**
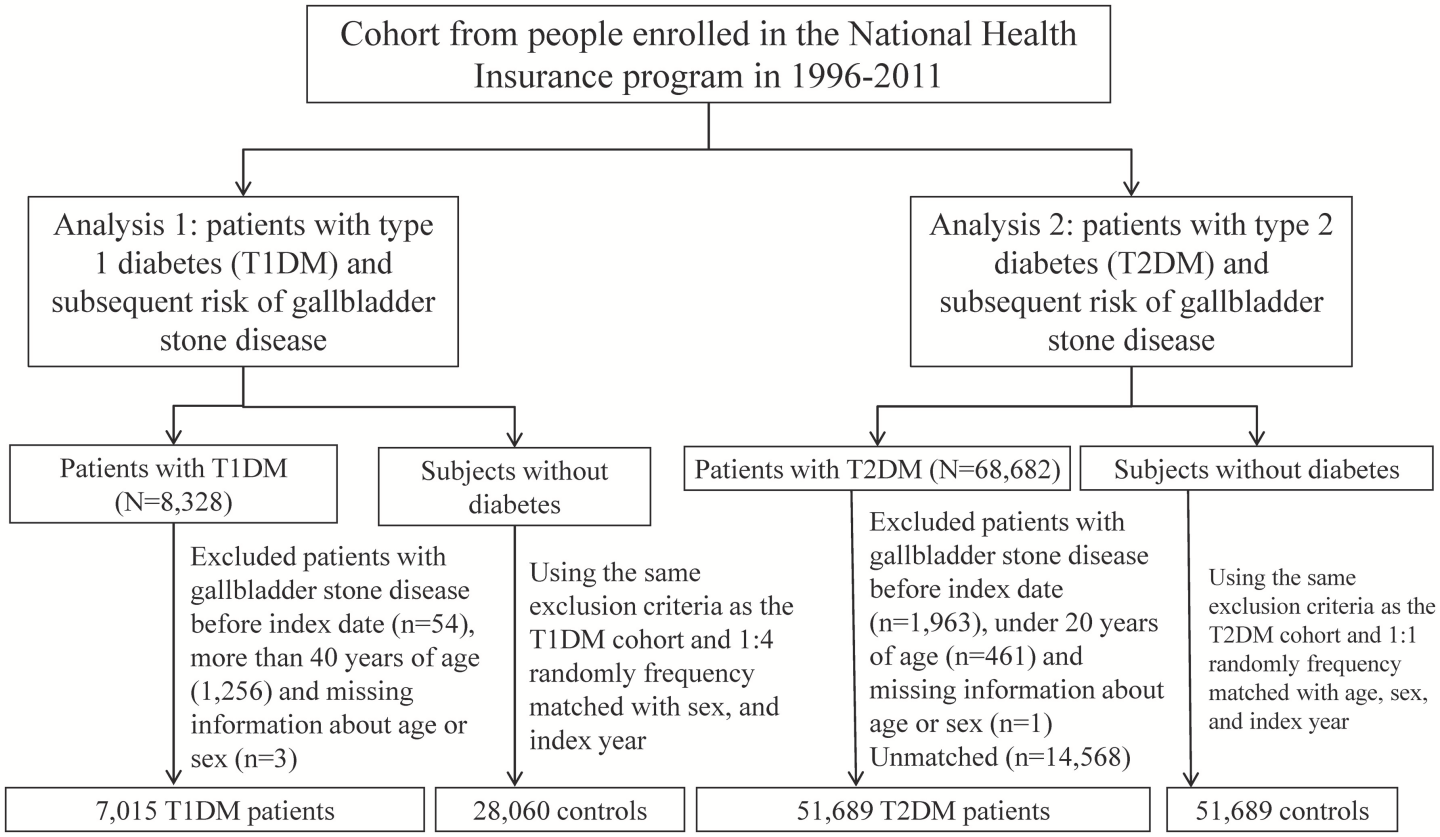
The selection process of the two study cohort groups to evaluate the association of T1DM and T2DM with the development of GSD.

We identified two study cohort groups to evaluate the association of T1DM and T2DM with the development of GSD from the (NHIRD), a database of Taiwan National Health Insurance (NHI) program (Figure 1) (19). The 2001 International Classification of Diseases, Ninth Revision, Clinical Modification (ICD-9-CM) was used for disease coding. The T1DM cohort and T2DM cohort were not similar in age distribution due to their different predilection of age for the onset of diabetes. Instead of comparing the risk of GSD between T1DM and T2DM, we individually created two matched control cohorts with T1DM and T2DM to compare the risk of T1DM and T2DM for the subsequent development of GSD with the control cohort, respectively.

The first group comprised patients with newly diagnosed T1DM (ICD-9-CM codes 250.0 × 1 and 250.0 × 3) who were aged ≤40 years based on the Registry of Catastrophic Illnesses Patient Database (RCIPD), a subset of NHIRD, between January 1, 1996, and December 31, 2011. The date of T1DM diagnosis was considered the index date. The non-diabetes cohort was frequency-matched in a 4:1 ratio according to sex, and the index year.

The second group comprised patients with newly diagnosed T2DM (ICD-9-CM codes 250.0 × 0 and 250.0 × 2) who were aged ≥20 years from the Longitudinal Health Insurance Database 2000 (LHID 2000), a subset of NHIRD, between January 1, 1996, and December 31, 2011. The date of T2DM diagnosis was also considered the index date. The non-diabetes cohort was frequency-matched in a 1:1 ratio according to age (at every 5-year interval), sex, and the index year.

Comorbidities included in our study were hypertension (ICD-9-CM codes 401-405), hyperlipidemia (ICD-9-CM codes 272), cirrhosis (ICD-9-CM codes 571 and A347), stroke (ICD-9-CM codes 430-438), coronary artery disease (CAD) (ICD-9-CM codes 410-414), chronic kidney disease (CKD) (ICD-9-CM codes 585, 586, 588.8, 588.9), alcohol-related illness (ICD-9-CM codes 291, 303, 305, 571.0, 571.1, 571.2, 571.3, 790.3, A215, and V11.3), and obesity (ICD-9-CM code 278). Furthermore, the number of parity was also considered in the analysis.

Patients with a history of GSD (ICD-9-CM codes 574.0, 574.1, 574.2, 574.6, 574.7, 574.8, and 574.9) or those with incomplete information on age or sex at the baseline were excluded from the cohorts. Each patient was examined from the index date until the development of GSD, withdrawal from the NHI program, or the end of 2011. The reasons for withdrawal included emigration from Taiwan or death during the follow-up period. Both cause-specific and non-cause-specific deaths were included in the analysis if available, and the deaths were censored when the causes were unknown. The index date of the control patients was matched according to the index year, month, and day of the case patients.

### Data availability statement

The dataset used in this study is held by the Taiwan Ministry of Health and Welfare (MOHW). The Ministry of Health and Welfare must approve our application to access this data. Any researcher interested in accessing this dataset can submit an application form to the Ministry of Health and Welfare requesting access. Please contact the staff of MOHW (Email: stcarolwu@mohw.gov.tw) for further assistance. Taiwan Ministry of Health and Welfare Address: No.488, Sec. 6, Zhongxiao E. Rd., Nangang Dist., Taipei City 115, Taiwan (R.O.C.). Phone: +886-2-8590-6848. All relevant data are within the paper.

### Ethics statement

The NHIRD encrypts patient personal information to protect privacy and provides researchers with anonymous identification numbers associated with relevant claims information, including sex, date of birth, medical services received, and prescriptions. This study was approved to fulfill the condition for exemption by the Institutional Review Board (IRB) of China Medical University (CMUH104-REC2-115-CR1). The IRB also specifically waived the consent requirement.

### Statistical analysis

The categorical variables, such as distributions of age, sex, comorbidities, and number of parity were compared between the case and control cohorts by using the chi-squared test. The continuous variables, such as the mean ages (standard deviations, SDs) and follow-up period, were compared between the case and control cohorts by using the Student *t*-test. The cumulative incidence of GSD and survival between the study cohorts was compared using the Kaplan-Meier method, and the differences were examined using the log-rank test. We expressed the incidence density rates of GSD by dividing the incidence of GSD by the number of person-years for each risk factor. The incidence density rates of GSD in patients with T1DM, T2DM, or without diabetes were stratified by age, sex, and comorbidities, respectively. We used univariable and multivariable Cox proportional hazard regression models to assess the diabetes-related risk of GSD. The hazard ratios (HRs) and 95% confidence intervals (CIs) were estimated using the Cox model, after adjustment for age, sex, comorbidities of hypertension, hyperlipidemia, cirrhosis, stroke, CAD, CKD, alcohol-related illness and obesity, and number of parity. The extensions of the standard univariable and multivariable Cox proportional hazard regression models were used in considering the death event as a competing event to estimate sub-HRs (SHRs) and 95% CIs. All the data were analyzed using SAS Version 9.4 (SAS Institute, Cary, NC, USA) software, and a two-tailed *P* value of < 0.05 was considered significant.

## Results

Table 1 presents the demography between patients with and without diabetes. In this study, the T1DM and non-diabetes cohorts included 7,015 and 28,060 patients, respectively. The mean ages of patients in the T1DM and non-diabetes cohorts were 18.9 ± 10.3 and 22.9 ± 10.3 years, respectively. Most patients with T1DM were younger than 20 years (56.9%) and were women (52.2%). The patients with T1DM had predispositions toward comorbidities, with hyperlipidemia (33.5%) and hypertension (13.4%) ranking as the top two comorbidities. In this study, the T2DM and non-diabetes cohorts included 51,689 patients each. The mean ages of patients in the T2DM and non-diabetes cohorts were 57.1 ± 13.2 and 56.4 ± 13.7 years, respectively. Most patients with T2DM were middle aged (41–60 years: 50.0%) and were men (54.2%). The T2DM patients similarly had a predilection toward comorbidities, with hypertension (72.4%), and hyperlipidemia (63.6%) ranking as the top two comorbidities.

**Table 1:** Characteristics of patients with and without diabetes.

	**Type 1 diabetes**					**Type 2 diabetes**				
	**No**		**Yes**			**No**		**Yes**	
	**(*****N*** = **28,060)**		**(*****N*** = **7,015)**			**(*****N*** = **51,689)**		**(*****N*** = **51,689)**	
	***n***	**%**	***n***	**%**	***p*****-value**	***n***	**%**	***n***	**%**	***p*****-value**
**Age, year**					0.001					0.99
≤ 20	11111	39.6	3989	56.9					
21–40	16949	60.4	3026	43.1		5012	9.70	5012	9.70
41–60						25821	50.0	25821	50.0
61–80						18805	36.4	18805	36.4
>80						2051	3.97	2051	3.97
Mean (SD)*	22.9	10.3	18.9	10.3	<0.001	56.4	13.7	57.1	13.2	0.001
**Sex**					0.99					0.99
Women	14648	52.2	3662	52.2		23685	45.8	23685	45.8
Men	13412	47.8	3353	47.8		28004	54.2	28004	54.2
**Comorbidity**
Hypertension	932	3.32	939	13.4	<0.001	21697	42.0	37399	72.4	<0.001
Hyperlipidemia	981	3.50	2350	33.5	<0.001	12873	24.9	32858	63.6	<0.001
Cirrhosis	74	0.26	755	10.8	<0.001	918	1.78	2067	4.00	<0.001
Stroke	98	0.35	96	1.37	<0.001	4169	8.07	8171	15.8	<0.001
CAD	260	0.93	192	2.74	<0.001	10908	21.1	17917	34.7	<0.001
Chronic kidney diseases	44	0.16	696	9.92	<0.001	1212	2.34	3367	6.51	<0.001
Alcohol-related illness	994	3.54	370	5.27	<0.001	2982	5.77	4652	9.00	<0.001
Obesity	514	1.83	220	3.14	<0.001	827	1.60	2477	4.79	<0.001
**Number of parity**					<0.001					<0.001
0	25062	89.3	6694	95.4		50242	97.2	50879	97.9
1	1524	5.43	168	2.39		871	1.69	660	1.28
2+	1474	5.25	153	2.18		576	1.11	450	0.87

*SD, standard deviation; CAD, coronary artery disease; Chi-squared test*;

* *t-test*.

**Figure 2:**
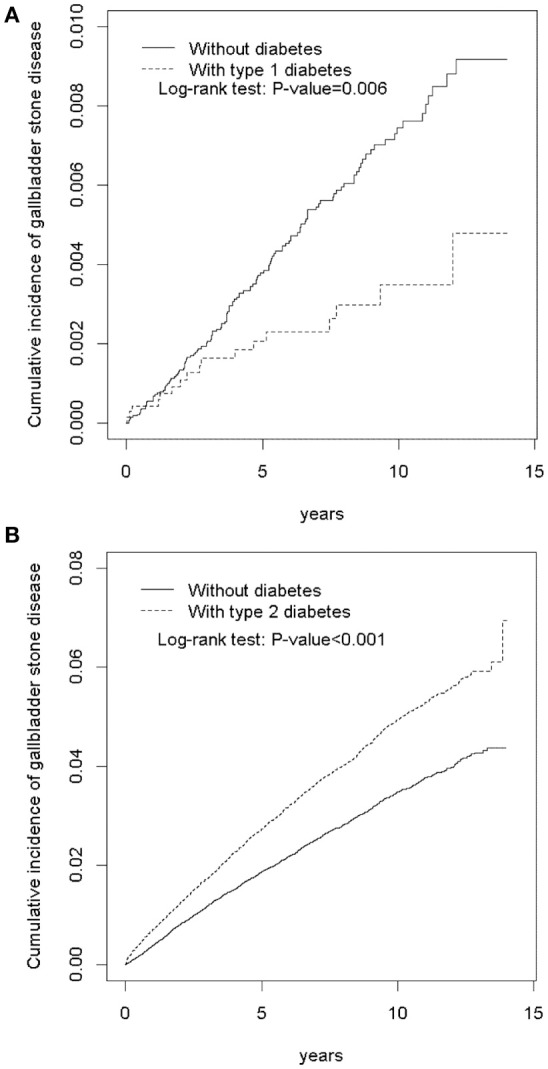
Cumulative incidence of gallbladder stone disease between patients with type 1 diabetes **(A)** and patients with type 2 diabetes **(B)**, and without diabetes.

Figure 2A indicates that the cumulative incidence of GSD was lower in the T1DM cohort than in the non-diabetes cohort (log-rank test, *P* < 0.001); the average follow-up duration was 6.56 ± 3.95 years for the T1DM cohort and 6.46 ± 3.95 years for the non-diabetes cohort. Figure 2B demonstrates that the cumulative incidence of GSD was greater in the T2DM cohort than in the non-diabetes cohort (log-rank test, *P* < 0.001); the average follow-up duration was 6.58 ± 3.92 years for the T2DM cohort and 6.33 ± 3.40 years for the non-diabetes cohort.

Table 2 shows the incidence and HRs of GSD in the T1DM or T2DM cohort compared with those in the control cohort. The overall incidence density rates of GSD in patients aged ≤ 40 years with T1DM and without diabetes were 3.70 and 7.39 per 10,000 person-years, respectively. Compared with patients without diabetes, those with T1DM had a decreased risk of GSD [adjusted HR (aHR) = 0.48, 95% CI = 0.25–0.92], after adjustment for age, sex, hypertension, hyperlipidemia, cirrhosis, stroke, CAD, CKD, alcohol-related illness, obesity, and number of parity. The overall incidence density rates of GSD in patients aged ≥20 years with T2DM and without diabetes were 51.6 and 33.5 per 10,000 person-years, respectively. Relative to patients without diabetes, those with T2DM were associated with an increased risk of GSD (aHR = 1.55, 95% CI = 1.41–1.69), after adjustment for age, sex, hypertension, hyperlipidemia, cirrhosis, stroke, CAD, CKD, alcohol-related illness, obesity, and number of parity.

**Table 2:** Incidence and HRs of gallbladder stone disease (GSD in type 1 diabetes cohort or type 2 diabetes cohort compared with the corresponding control cohort.

	**Type 1 diabetes**		**Type 2 diabetes**	
	**No**	**Yes**	**No**	**Yes**
	**(*N* = 28,060)**	**(*N* = 7,015)**	**(*N* = 51,689)**	**(*N* = 51,689)**
Person-years	181,341	45,985	453,383	435,806
Follow-up time (y) Median ± (SD)	6.46 ± 3.95	6.56 ± 3.95	6.33 ± 3.40	6.58 ± 3.92
**GSD**				
Event	134	17	1611	2249
Rate^#^	7.39	3.70	35.5	51.6
Crude HR (95% CI)	1 (Reference)	0.50 (0.30, 0.83)**	1 (Reference)	1.45 (1.36, 1.54)***
Adjusted HR (95% CI)	1 (Reference)	0.48 (0.25, 0.92)*	1 (Reference)	1.55 (1.41, 1.69)***

*Rate#, incidence rate, per 10,000 person-years; Crude HR, relative hazard ratio; SD, standard deviation; 95% CI, 95% confidence interval; CAD, coronary artery disease; Adjusted HR, multivariate analysis including age, sex, hypertension, hyperlipidemia, cirrhosis, stroke, CAD, chronic kidney diseases, alcohol-related illness, obesity, and number of parity*.

* *P < 0.05*,

** *P < 0.01*,

*** *P < 0.001*.

Table 3 presents a comparison of the GSD incidence densities between patients with T1DM, T2DM, and without diabetes in terms of demographic characteristics and comorbidities. The age-specific relative risk of GSD in the T1DM cohort was lower than that in the non-diabetes cohort for patients aged 21–40 years (aHR = 0.39, 95% CI = 0.19–0.81). The relative risk of GSD in the T1DM cohort was lower than that in the non-diabetes cohort for both sexes (women: aHR = 0.46, 95% CI = 0.19–1.08; men: aHR = 0.46, 95% CI = 0.16–1.27) and for patients without comorbidities (aHR = 0.33, 95% CI = 0.08–1.34) or with comorbidities (aHR = 0.56, 95% CI = 0.30–1.04), but there was no statistical significance. Except for age group >80 years, the relative risk of GSD in the T2DM cohort was statistically greater than that in the non-diabetes cohort for each age group, both sexes, and patients without comorbidities or with comorbidities.

**Table 3:** Incidence and hazard ratios of gallbladder stone disease measured by age, sex, and comorbidity in type 1 diabetes or type 2 diabetes cohort compared with the corresponding control cohort.

	**Type 1 diabetes**					**Type 2 diabetes**			
	**No (*****N*** = **28,060)**		**Yes (*****N*** = **7,015)**			**No (*****N*** = **51,689)**		**Yes (*****N*** = **51,689)**	
**Variables**	**Event**	**Rate**^#^	**Event**	**Rate**^#^	**Adjusted HR** **(95% CI)**	**Event**	**Rate**^#^	**Event**	**Rate**^#^	**Adjusted HR (95% CI)**
**AGE, YEARS**										
≤ 20	15	1.88	3	1.12	0.42 (0.09, 1.96)				
21–40	119	11.7	14	7.30	0.39 (0.19, 0.81)*	31	9.86	89	28.2	2.82 (1.75, 4.57)***
41–60						416	25.0	611	37.1	1.69 (1.46, 1.96)***
61–80						545	44.9	713	60.5	1.44 (1.28, 1.63)***
> 80						52	63.0	53	65.0	1.08 (0.73, 1.61)
**SEX**										
Women	80	8.40	11	4.57	0.46 (0.19, 1.08)	877	39.8	1189	56.3	1.53 (1.35, 1.73)***
Men	54	6.27	6	2.74	0.46 (0.16, 1.27)	734	31.5	1060	47.2	1.56 (1.38, 1.78)***
**COMORBIDITY**^§^										
No	81	5.56	2	0.96	0.33 (0.08, 1.34)	296	22.3	130	61.6	2.72 (2.21, 3.34)***
Yes	53	14.9	15	5.96	0.56 (0.30, 1.04)	748	38.4	1336	44.4	1.24 (1.13, 1.36)***

*Rate#, incidence rate, per 10,000 person-years; Crude HR, relative hazard ratio; 95% CI, 95% confidence interval. Adjusted HR: multivariate analysis including age, sex, hypertension, hyperlipidemia, cirrhosis, stroke, coronary artery disease (CAD), chronic kidney diseases, alcohol-related illness, obesity, and number of parity; Comorbidity^§^: Patients with any one of the comorbidities (including hypertension, hyperlipidemia, cirrhosis, stroke, CAD, chronic kidney diseases, and alcohol-related illness) were classified as the comorbidity group*.

* P < 0.05;

*** *P < 0.001*.

After considering the competing risk of death, we observed that the T1DM cohort consistently had a significantly lower risk of GSD than did the non-diabetes cohort (adjusted SHR = 0.49, 95% CI = 0.25–0.93) (Table 4). However, the T2DM cohort had a significantly greater risk of GSD than did the non-diabetes cohort (adjusted SHR = 1.69, 95% CI = 1.55–1.85).

**Table 4:** Incidence and SHRs of gallbladder stone disease in type 1 diabetes or type 2 diabetes cohort compared with the corresponding control cohort, based on the competing risk regression.

	**Type 1 diabetes**		**Type 2 diabetes**	
	**No**	**Yes**	**No**	**Yes**
	**(*N* = 28,060)**	**(*N* = 7,015)**	**(*N* = 51,689)**	**(*N* = 51,689)**
Crude SHR (95% CI)	1 (Reference)	0.68 (0.41, 1.13)	1 (Reference)	1.65 (1.54, 1.76)***
Adjusted SHR(95% CI)	1 (Reference)	0.49 (0.25, 0.93)*	1 (Reference)	1.69 (1.55, 1.85)***

Crude SHR, relative subhazard ratio; Adjusted SHR†: multivariate analysis including age, sex, hypertension, hyperlipidemia, cirrhosis, stroke, coronary artery disease, chronic kidney diseases, alcohol-related illness, obesity, and number of parity;

* *P < 0.05*,

*** *P < 0.001*.

## Discussion

Our findings are consistent with those in the literature in revealing that most patients with T1DM were women (52.2%) and most patients with T2DM were men (54.2%); furthermore, most patients with T1DM were younger than 20 years (56.9%) and most patients with T2DM were middle aged (50–64 years: 40.5%) (11, 19). A slight men predominance was reported in countries with a high T1DM incidence, and the opposite was noted in countries with a low T1DM incidence, whereas the difference of sex predilection was small in T1DM (11). Most cases of T1DM were diagnosed between birth and 14 years of age, but the incidence steadily increased up to 10–15 years old (19). The incidence of T2DM in Taiwan was reported to be higher in men. In addition, the age of onset of T2DM became younger and was predominantly in the age bracket of 50–64 years due to the increasing prevalence of metabolic syndrome, a sedentary lifestyle, and westernized dietary habits (20).

Consistent with the literature, hypertension and hyperlipidemia were the common comorbidities associated with T1DM or T2 DM in the current study (Table 1) (21, 22). T1DM was reported to be related to arterial stiffness due to chronic inflammation and its genetic polymorphisms to atherosclerosis (23). Reports have revealed that hyperglycemia, hypoglycemia, altered fat distribution, thrombosis, and increased adipokines in T1DM can cause chronic vascular inflammation. As a result of the presence of haptoglobin 2-2, with reduced antioxidant capacity and impaired reverse cholesterol transport, patients with T1DM are also predisposed to the development of cardiovascular disease. In addition to common metabolic disorders shared by hypertension and T2DM, T2DM predisposes a patient to hypertension through IR, with impaired fibrinolysis and chronic inflammation, and through high insulin level, with increased arterial stiffness, vascular volume, and sympathetic tone (24). Additionally, the liver overproduces very low-density lipoprotein cholesterol and the intestines overproduce chylomicrons in patients with insulin deficiency (T1DM) or IR (T2DM) (25).

Our results reveal an inverse association between T1DM and GSD, after adjustment for age, sex, and comorbidities of hypertension, hyperlipidemia, cirrhosis, stroke, CAD, CKD, and alcohol-related illness (Table 2). One could argue that the follow-up duration was not sufficient (6.56 ± 3.95 years) for the T1DM cohort and 6.46 ± 3.95 years for the non-diabetes cohort) and the mean age of the cohorts was relatively young (with the mean ages of patients in the T1DM and non-diabetes cohorts being 18.9 ± 10.3 and 22.9 ± 10.3 years) to support the inverse association between T1DM and GSD. However, we observed an inverse association— rather than no association— and the reduced risk of GSD was more obvious in the age group of 21–40 years, instead of younger patients aged ≤20 years. After considering the competing risk of death, we observed that the T1DM cohort consistently had a significantly lower risk of GSD than did the non-diabetes cohort. Furthermore, the relative risk of GSD in the T1DM cohort decreased with the follow-up duration after T1DM diagnosis, even though the follow-up duration was longer in the T1DM cohort. These findings support a decreased risk of GSD after T1DM diagnosis.

Our results reveal a strong association between T2DM and GSD, after adjustment for age, sex, and comorbidities of hypertension, hyperlipidemia, cirrhosis, stroke, CAD, CKD, and alcohol-related illness (Table 2). The relative risk of GSD was higher in the T2DM cohort than in the non-diabetes cohort for each age group, sex, and patients with or without comorbidities (Table 3). After considering the competing risk of death, we noted that the T2DM cohort consistently had a greater risk of GSD than did the non-diabetes cohort. Moreover, the relative risk of GSD in the T2DM cohort increased with the follow-up duration after T2DM diagnosis. These findings support an increased risk of GSD after T2DM diagnosis, although we could not ascertain the causal relationship between T2DM and GSD in this observational study.

The possible pathophysiological mechanisms underlying the development of GSD after the diagnosis of T2DM are outlined as follows: The first mechanism is increased plasma insulin level, resulting from IR, which predisposes a patient to bile supersaturation by decreasing bile salt secretion to cause bile supersaturation and increased mucus production by inducing gallbladder inflammation (13, 26). One animal study of mice with increased plasma insulin secretion due to hepatic IR has demonstrated that insulin itself will predispose to cholesterol gallstone formation due to an increase in the secretion of biliary cholesterol and lithogenic bile salt (27). The second mechanism is impaired gallbladder emptying due to autonomic neuropathy (28). The third mechanism is impaired cholecystokinin (CCK) secretion in jejunum and reduced sensitivity to CCK in T2DM, and the severity aggravates for the patients with autonomic neuropathy (14). The fourth mechanism is impaired gallbladder emptying due to hyperglycemia, which also co-exists with T1DM (17). However, the contribution of hyperglycemia on the development of GSD remains disputed since the literature reported that no difference was found in glycemic control (HBA1C) between T2DM with and without GSD and no association between T1DM and GSD was found despite the majority of T1DM had poor glycemic control (16, 29). The final mechanism is the coexistence of metabolic risk factors, which will enhance the biliary supersaturation, in T2DM and GSD. However, positive association between T2DM and GSD and inverse association between T1DM and GSD were consistently found in our study after controlling the potential confounding factors. The possible pathophysiological mechanisms underlying the decreased risk of GSD after the diagnosis of T1DM are outlined as follows: The first mechanism is the relatively rare coexistence of metabolic risk factors in T1DM and GSD. The second mechanism is the relatively rare incidence of neuropathy, which can impair the gallbladder emptying, in T1DM (30). The final mechanism is insulin deficiency, which has been suggested to be the main protective factor for GSD, although the definite mechanism remains unknown (16, 31). However, insulin *per se* will increase the secretion of biliary cholesterol and lithogenic bile salt as mentioned in the literature (27). Some detrimental factors for the development of GSD in T2DM, such as impaired gallbladder emptying due to hyperglycemia and coexistence of metabolic risk factors, may also be found in T1DM. Although the plasma insulin level may be the important determinant for the development of GSD and can explain the outcome difference between T1DM and T2DM, it requires more studies to clarify the summation effect of the detrimental and protective factors for the development of GSD in T1DM (27). Our findings support that age is an important determinant for the development of GSD since the rate of GSD increases with incremental age. It may be too early for the individuals aged ≤ 20 to develop GSD, and the protective effect of T1DM on the development of GSD cannot not be observed until after age 20. However, it needs more studies to ascertain whether the protective effect of T1DM on the development of GSD can overpass the detrimental effect of aging for the elderly.

The strengths of this study are described as follows. First, this is the largest retrospective population-based cohort study to investigate the association between T1DM and GSD, which provides a high statistical power and generalizability of our findings in Taiwan. The flow chart (the Figure 1) has showed the selection procedure of studied participants. Between January 1, 2000, and December 31, 2010, all the subjects with diabetes diagnosed from the database of NHIRD were followed forward retrospectively. The incidence of GSD for T1DM was never reported in Taiwan, although its incidence was reported to be comparable to that for T2DM in Italy (18). Second, to our knowledge, this study has provided the longest follow-up duration to assess the association between T1DM and GSD (15, 31). Moreover, the ages of the enrolled patients with T1DM are the oldest (40 years old) in the literature. Third, through utilizing the NHIRD, a longitudinal database with a 12-year observation period for residents of Taiwan from the NHI program covering more than 99.6% of the 23.74 million residents of Taiwan, this study represents the actual association between diabetes and GSD in Taiwan.

This study also has some limitations. First, the data on body mass index, lifestyle and dietary habits are not available in the NHIRD. However, we substituted the diagnosis of obesity for body mass index and alcohol-related illness for alcohol consumption habits, respectively. Second, we could not individually validate the medical records. Nevertheless, all insurance claims should abide by the standard diagnosis criteria to avoid statutory sanctions and financial penalties by the NIH program authorities. Furthermore, the multivariable Cox proportional hazards regression model revealed a consistent inverse association between T1DM and GSD and a positive association between T2DM and GSD. Third, more time may be required to provide a follow-up on the development of GSD. However, this study has provided the longest follow-up of patients with T1DM, and the ages of the enrolled T1DM patients are up to middle age. Finally, we could not ascertain the definite causal relationship between diabetes and GSD, either positive or inverse association, in this observational study.

In conclusion, our population-based cohort study indicates a strong association between T2DM and GSD. However, an inverse relationship exists between T1DM and GSD in patients aged between 20 and 40 years. Further study is required to clarify the definite pathogenesis of diabetes to the development of GSD.

## Strengths and limitations of this study

1. This is the largest population-based cohort study with a 12-year period of follow-up by using of a single administrative database to investigate the association between T1DM and GSD, the large sample size provides a high statistical power and generalizability of our findings in Taiwan.
2. The data on lifestyle and dietary habits are not available in the NHIRD.
3. We could not ascertain the definite causal relationship between diabetes and GSD in this observational study.

## Author contributions

C-HC and C-HK: conceptualization, investigation; C-HC, C-LL, and C-HK: data curation, formal analysis, validation and visualization.; C-YH: funding acquisition; C-LL and C-HK: methodology, supervision; C-YH and C-HK: project administration; C-HC, C-YH, and C-HK: resources; C-LL, C-YH, and C-HK: software; All authors: writing (original draft preparation) and writing (review and editing).

### Conflict of interest statement

The authors declare that the research was conducted in the absence of any commercial or financial relationships that could be construed as a potential conflict of interest.

## Abbreviations

T2DM: type 2 diabetes
GSD: gallbladder stone disease
T1DM: type 1 diabetes
aHR: adjusted hazard ratio
CI: confidence interval
IR: insulin resistance
NHI: National Health Insurance
NHIRD: National Health Insurance Research Database
NHRI: National Health Research Institute
ICD-9-CM, International Classification of Diseases, Ninth Edition: Clinical Modification
CAD: coronary artery disease
CKD: chronic kidney disease
SHR: subhazard ratio
CCK: cholecystokinin.
